# Time to Change’s social marketing campaign for a new target population: results from 2017 to 2019

**DOI:** 10.1186/s12888-019-2415-x

**Published:** 2019-12-27

**Authors:** Clara González-Sanguino, Laura C. Potts, Maria Milenova, Claire Henderson

**Affiliations:** 1 Clinical Psychology Department, School of Psychology, University Complutense of Madrid, Madrid, Spain; 2 Statistician, Biostatistics and Health Informatics Department,Institute of Psychiatry, Psychology and Neuroscience, King’s College London, London, UK; 3 Centre for Global Mental Health, Institute of Psychiatry, Psychology and Neuroscience, King’s College London, London, UK; 4 Institute of Psychiatry, Psychology and Neuroscience, King’s College London, London, UK

**Keywords:** Mental illness stigma, Global mental health, Social marketing campaign

## Abstract

**Background:**

Since 2009 Time to Change has included among its strategies a social marketing campaign to tackle the stigma surrounding mental health problems. At the start of its third phase (2016–2021) the target group of the campaign was kept as people aged between mid-twenties and mid-forties but changed to middle-low income groups and the content was focused on men.

**Methods:**

Participants (*n* = 3700) were recruited through an online market research panel, before and after each burst of the campaign. They completed an online questionnaire evaluating knowledge (Mental Health Knowledge Schedule, MAKS); attitudes (Community Attitudes toward Mental Illness, CAMI); and desire for social distance (Intended Behaviour subscale of the Reported and Intended Behaviour Scale, RIBS). Socio-demographic data and awareness of the campaign were also collected.

**Results:**

For each of the 3 bursts, significant pre-post awareness differences were found (OR = 2.83, CI = 1.90–4.20, *p* < 0.001; OR = 1.72, CI = 1.22–2.42, *p* = 0.002; OR = 1.41, CI = 1.01–1.97, *p* = 0.043),

and awareness at the end of the third burst was 33%. Demographic factors associated with awareness for one or more bursts included having children, familiarity with mental illness, male sex, being Black, Asian or other ethnic minorities and living in London or the East Midlands regions. An improvement across bursts in the “living with” subscale item of the RIBS, and in the “recover” and “advice to a friend” MAKS items were found. Familiarity with mental illness had the strongest association with all outcome measures, while the awareness of the campaign was also related with higher scores in MAKS and RIBS.

**Conclusions:**

These interim results suggest that the campaign is reaching and having an impact on its new target audience to a similar extent as did the TTC phase 1 campaign. While over the course of TTC we have found no evidence that demographic differences in stigma have widened, and indeed those by age group and region of England have narrowed, those for socioeconomic status, ethnicity and sex have so far remained unchanged. By targeting a lower socioeconomic group and creating relatively greater awareness among men and in Black and ethnic minority groups, the campaign is showing the potential to address these persistent differences in stigma.

## Background

The stigma associated with mental illness involves negative thoughts, emotions and behaviours towards these people [1] who must not only face their psychological problems, but also the social discrimination caused by this phenomenon producing a restriction of rights and opportunities, leading to rejection in the social environment and favouring exclusion [1], social inequality and discrimination when obtaining a job or housing [2, 3].

Several initiatives have been launched to combat this phenomenon in various countries [4–7], among them the Time to Change programme in England [8] (https://www.time-to-change.org.uk/). Since 2009 it has aimed to be a growing social movement to change the way people think and act about mental health problems, raising awareness of what common mental health problems are, and letting the public know what they can do to help.

One of the main components of Time to Change is the social marketing campaign. Used to reach the public, its purpose is to tackle stigma surrounding mental health by demonstrating how common these problems are in all samples of society and giving people the tools to step in and support someone who is struggling. Empirical evidence exists from the work on intergroup contact to improve both knowledge, attitude and intended behaviour.

Both mass media and social media have been well documented as an immensely powerful source of social influence and intend to reach large numbers of people [9, 10]. From its launch to 2016 (Time to Change phases 1 and 2) the campaign was aimed at people aged between mid-twenties to mid-forties, from middle-income groups.

The evaluation of Time To Change is based on the theory that considers stigma as a lack of knowledge about mental illness; negative attitudes towards people with mental illness; and discriminatory behaviour towards them [11]. The results for phases 1 and 2 show an association between awareness of the campaign and each of knowledge, attitudes and desire for social distance, and improvements over the course of phases 1 and 2 in these outcomes. The changes during phases 1 and 2 were quite gradual; those first observed were in domains of mental health related knowledge and intended behaviour, followed by changes in the total scores of each of knowledge, attitudes and intended behaviour [12, 13].

Time to Change is currently in its third phase of delivery. While the target group age of 25–45 is unchanged the target income group is now low to middle instead of middle, and the content focusses on men to try to attract their attention. Also, parents were included as a target. This change aims to address inequalities in demographic groups in stigma, due to persistent differences by income group and sex which have neither widened nor narrowed over the course of Time to Change [14].

The objectives of this study are to examine: awareness of Time to Change over the first three bursts of the Phase 3 campaign in samples of the new target population, and factors associated with awareness; changes in outcomes of stigma related knowledge, attitudes and desire for social distance over this time period; and the relationship between awareness of the campaign and the outcomes.

## Methods

### Design

Participants in the demographic groups targeted by the social marking campaign were recruited via an online market research panel before and after each of the three bursts of the campaign (each survey wave using different participants). A burst of the campaign is defined as a process of media buying over a few weeks aimed at exposing the programme to the largest audience possible. On line data collection is used as this reduces the cost per respondent and because previous work suggests that behavioural intentions towards people with mental health problems may be better assessed using online self-complete methods rather than in-person interviews [15]. Quotas were set for each type of media used to enhance the likelihood that survey participants were exposed to campaign materials. Online panel interviews were performed pre and post each of the three bursts of campaign activity. Quotas were also set to include equal distributions of age, sex, and socio-economic status and the sample was designed to be geographically representative of the population in England. Ethnic minority participants were oversampled.

#### Intervention: the social marketing campaign

The social marketing campaign covered by this evaluation is comprised by three bursts of multimedia activity, each lasting several weeks, with one in April of 2017 and two in February of 2018 and 2019. The campaign media targeted men and women in their mid-twenties to mid- forties in an overlapping income group, but consisting of lower social classes than in previous phases: C1: lower-middle class (Supervisory, clerical and junior managerial, administrative and professional); C2: skilled working class (Skilled manual workers); D: skilled manual occupation (Semi-skilled and unskilled manual workers); and more directly towards men, as compared to B (Intermediate managerial, administrative or professional), C1 and C2 in phases 1 and 2. In addition, activities directed at parents were introduced with the aim of facilitating open conversations, to make talking about mental health as every day and ordinary as other parent/child conversations.

The campaign included the use of social media such as Facebook, Twitter, Instagram and Snapchat; radio adverts across several stations, digital content platforms; partnership with Joe Media [a media company established in the United Kingdom (UK) in 2015 specialised in sport, politics, lifestyle and pop culture] and beer mats and washroom posters in pubs. In Time to Change phase 1 the focus was on knowledge and attitudes; during phase 2 and currently in phase 3 the focus is on behaviour change. The previous key messages of the campaign to encourage supportive contact were reworked for this target group. In the first two bursts the campaign encouraged people to ‘be in their mate’s corner’, harnessing the power of friendship and humour to reach a more detached audience. The third campaign burst developed this idea further, encouraging people to ‘ask twice’ if they feel like someone they know is acting differently. Hence, the campaign promotes empathy towards people with a mental health problem as a key mediator of the effect of contact on prejudice [16] while encouraging people to maintain direct contact [17] (as opposed to social distancing). In the process, the advertising provides parasocial (virtual) contact [17] and promotes imagined contact [18]. For parents a specific section with parent information was included in the Time To Change website; and short films were used in public relations and social media. This clear call to action provides the target audience with practical advice about starting a conversation, something for which there is evidence in terms of suicide prevention [19].

### Instruments

#### Knowledge

Mental health-related knowledge was measured by the Mental Health Knowledge Schedule (MAKS) [20]. The MAKS comprises six items covering stigma-related mental health knowledge areas [20]: help seeking, recognition, support, employment, treatment, and recover, and six items that enquire about classification of various conditions as mental illnesses [21]. Each item is scored on a 5-point Likert scale, from 5 = ‘strongly agree’ to 1 = ‘strongly disagree’. The total score is calculated by adding together the response values of each item, and a higher score indicated greater knowledge.

#### Attitudes

Attitudes towards mental illness were assessed based on the 12 version item of the Community Attitudes toward the Mentally Ill Scale (CAMI) [22], previously used in Time To Change campaign evaluation [12] and in the Health Survey for England [23]. Each item is scored on a 5-point Likert scale, from 5 = ‘strongly agree’ to 1 = ‘strongly disagree’. The total score is calculated by adding together each single item, and higher score indicates more positive attitudes.

#### Desire for social distance

The desire for social distance (the level of intended future contact with people with mental health problems) was measured by the Intended Behaviour subscale of the Reported and Intended Behaviour Scale (RIBS) [24]. The RIBS consist of four domains (living with, working with, living nearby, and continuing a relationship with someone with a mental health problem) and assesses reported and the intended behaviour in each domain. In this study, only intended behaviour was evaluated. Each item is scored on a 5-point Likert scale, from 1 = ‘strongly disagree to engage in the stated behaviour’ to 5 = ‘strongly agree with engaging in the stated behaviour’. The total score is calculated by adding together each single item, and higher score indicated higher willingness to engage in the behaviour.

#### Campaign awareness

Prompted campaign awareness was assessed for each type of media and / or activity used by Time to Change. Individuals who reported seeing any of the advertisements were categorised as ‘campaign aware’ while those who responded ‘no’ or ‘don’t know’ were categorised as ‘not campaign aware’. Campaign awareness associated with the post-burst stage pertains to awareness of the specific media activity immediately preceding the survey, while awareness during the pre-stage refers to the recall of the media used in the previous campaign burst.

The assessment of the first pre stage used materials from phase 2 of Time to Change, and as awareness as assessed at each point comprises unprompted awareness as well as prompted awareness (i.e. using materials from the last campaign) it includes awareness of any previous TTC activity. There were no other campaigns to reduce stigma or increase mental health literacy during this period as the only other such campaign, Heads Together, had finished before the first burst of phase 3.

#### Social contact

Social contact with someone with a mental health problem was assessed by asking the following question: *Who is the person closest to you who has or has had some kind of mental health problem?* Scoring the answers in the following categories: self, immediate family (spouse/sister/brother/parents …), one of your children, partner (living with you), partner (not living with you), other family (uncle/aunt/cousin/grandparent …

), friend, acquaintance, work colleague, neighbours, ex-partner, no-one known. For more simplicity in the analysis the categories were reduced to three: no-one-known, self, other.

### Statistical analysis

All analyses were weighted to reflect population characteristics in England. Survey weights were developed using prevalence rates of ethnicity with geographic region from the UK Government’s Office for National Statistics. All models were adjusted for the impact of the “Burst” as well as main relevant socio-demographic characteristics identified from the literature in the field [i.e., gender; age; ethnicity; socioeconomic group; geographic region; marital status; having children; working status; degree of familiarity with mental illness (Categorized as me/other/no-one-known answering the question: Who is the person closest to you who has or has had some mental illness?).

Descriptive statistics for participant demographics were calculated and presented using unweighted frequency and weighted percentage/mean/standard deviation.

Adjusted logistic regression models were used to analyse campaign awareness. To examine whether there was a consistent pre/post effect, we included a variable indicating whether the assessment occurred before or after the burst of media (pre vs. post). We also investigated factors significantly associated with campaign awareness where the following independent variables were entered into the model: ethnicity (categorical: White, Asian, Black, Mixed or Other), gender, age (categorical: 25–29, 30–34, 35–39, 40–45), marital status (married: yes/no), having children (children: yes/no) employment status (categorical: employed (full or part-time employment), not working (unemployed or retired), student), socioeconomic group (categorical: lower middle class C1, skilled working class C2, semi-skilled and unskilled manual workers D), geographic region (categorical: Yorkshire and Humber, North East, North West, East Midlands, West Midlands, East of England, London, South East, South West) and social contact (categorical: having a mental health problem oneself, knowing someone with a mental health problem or not knowing anyone with a mental health problem).

Multivariable linear regression models were used to analyse the total MAKS, CAMI and RIBS scores. A pre/post effect for each outcome measure was investigated as described above. Multivariable logistic regression models estimated the odds of responding positively (i.e., agree strongly or agree slightly) to each of the MAKS and RIBS items. All items were coded so that agreement summarised a less stigmatising response. Presence of a long-term trend was examined by including campaign burst as a covariate in the model for the total score of MAKS, CAMI and RIBS, and for each item of the MAKS and RIBS scales.

The relationship between each of the outcome measures (CAMI, MAKS, RIBS) with campaign awareness was assessed by including the campaign awareness variable into the adjusted linear regression model. This will also inform us of factors associated with each outcome measure.

## Results

### Target population

3700 persons were interviewed between April 2017 and February 2019. The average age of the sample was 35.8 years, 51.8% were women, 44.8% lower-middle class (C1), 86.0% working at the time of the interview and 73.5% white. More details of the sample can be seen in Table 1.

**Table 1:** Participant’s socio-demographic characteristics, un-weighted frequency and weightedpercentages (*n* = 3700)

Demographic characteristic	N (%)
Gender, Female *n* (%)	1892 (51.82)
Age, mean (SD)	35.77 (5.68)
Age group	639 (17.10)
25–29	880 (24.42)
30–34	1060 (29.04)
35–39	1121 (29.44)
40–45	
Socioeconomic status, *n* (%)	
C1, lower middle class	1618 (44.84)
C2, skilled working class	1144 (29.89)
D, semi-skilled and unskilled manual workers	938 (25.27)
Employment status, *n* (%)	
Working	3209 (86.05)
Student	22 (0.74)
Not working	469 (13.2)
Married, yes, *n* (%)	2564 (69.69)
Children, yes, *n* (%)	2079 (57.49)
Ethnicity, *n* (%)	
Black	102 (4.71)
White	3140 (73.55)
Asian	368 (17.56)
Mixed	76 (3.66)
Other	14 (0.53)
Region	
North East	223 (6.51)
North West	555 (18.57)
Yorkshire & Humberside	416 (12.04)
East Midlands	361 (10.27)
West Midlands	398 (10.49)
East of England	398 (10.07)
London	538 (13.04)
South East	561 (14.44)
South West	250 (4.58)
Who is the person closest to you who has or has had some	
mental illness?	
No-one-known	1844 (49.45)
Self	384 (9.72)
Other	1472 (40.82)

### Campaign awareness

For each of the three bursts, significant pre-post awareness differences were found (OR = 2.83, CI = 1.90 to 4.20, *p* < 0.001; OR = 1.72, CI = 1.22 to 2.42, *p* = 0.002; OR = 1.41, CI = 1.01 to 1.97, *p* = 0.043), with similar levels of post-burst awareness of 33, 34 and 33% respectively.

#### Factors associated with campaign awareness

Characteristics significantly associated with campaign awareness in the first burst were being aged between 30 and 34 (OR = 1.59, CI = 1.09 to 2.31; *p* = 0.016) as compared to aged 40–45, being Asian (OR = 1.95, CI = 1.30 to 2.92; *p* = 0.001), knowing someone with a mental health problem (OR = 1.96, CI = 1.45 to 2.64; *p* < 0.001) and having children (OR = 1.49, CI = 1.06 to 2.09; *p* = 0.021). In the second burst, the factors associated with campaign awareness were being Asian (OR = 1.60, CI = 1.04 to 2.48; *p* = 0.033) as compared to White, being male (OR = 0.74, CI = 0.55 to 0.99; *p* = 0.047), having children (OR = 1.47, CI = 1.05 to 2.06; *p* = 0.025), having or having had a mental health problem (OR = 2.40, CI = 1.46 to 3.93; *p* = 0.001) and knowing someone with a mental health problem (OR = 2.10, CI = 1.57 to 2.82; *p* < 0.001). Finally, for the third burst, characteristics significantly associated with campaign awareness include male sex (OR = 0.62, CI = 0.46 to 0.84; *p* = 0.002), having children (OR = 1.82, CI = 1.31 to 2.53; *p* < 0.001), knowing someone with a mental health problem (OR = 1.78, CI = 1.30 to 2.42; *p* < 0.001), being Black or other ethnicity (OR = 4.51, CI = 1.67 to 12.17; *p* = 0.003; OR = 12.53, CI = 1.52 to 103.03; *p* = 0.019) and being from London (OR = 2.06, CI = 1.17 to 3.64; *p* = 0.013) as compared to Yorkshire and Humber.

Results of the regression to explore factors associated with campaign awareness, including reference categories, can be seen in Table 2.

**Table 2:** Results of the multivariate logistic regression models to explore factors associated with campaign awareness

	Burst 1 April 2017 (*n* = 1349)		Burst 2 February 2018 (*n* = 1179)		Burst 3 February 2019 (*n* = 1169)	
	OR (95% CI)	*p* value	OR (95% CI)	p value	OR (95% CI)	p value
Age						
25–29	1.29 (0.85–1.97)	0.235	1.07 (0.70–1.64)	0.750	1.13 (0.72–1.79)	0.592
30–34	1.59 (1.09–2.31)	0.016	1.02 (0.69–1.51)	0.907	1.09 (0.72–1.63)	0.684
35–39	1.10 (0.75–1.62)	0.624	1.31 (0.91–1.90)	0.148	1.05 (0.73–1.50)	0.801
40–45 (ref)						
Gender						
Female	0.87 (0.65–1.17)	0.350	0.74 (0.55–0.99)	0.047	0.62 (0.46–0.84)	0.002
Male (ref)						
Ethnicity						
Black	1.68 (0.89–3.17)	0.109	2.09 (0.88–4.97)	0.094	4.51 (1.67–12.17)	0.003
Asian	1.95 (1.30–2.92)	0.001	1.60 (1.04–2.48)	0.033	1.24 (0.76–2.03)	0.389
Mixed	0.94 (0.36–2.43)	0.895	1.73 (0.76–3.94)	0.191	2.24 (0.77–6.50)	0.137
Other	2.80 (0.39–20.14)	0.305			12.53 (1.52–103.03)	0.019
White (ref)						
Socioeconomic status						
C2, skilled working class	0.92 (0.65–1.29)	0.624	1.32 (0.95–1.85)	0.099	1.09 (0.77–1.53)	0.635
D, working class	1.09 (0.77–1.55)	0.629	0.95 (0.65–1.38)	0.772	0.93 (0.64–1.36)	0.723
C1, low-middle class (ref)	–	–	–	–	–	
Married						
Yes	1.13 (0.79–1.61)	0.504	1.08 (0.76–1.54)	0.663	1.27 (0.88–1.83)	0.195
No (ref)						
Children						
Yes	1.49 (1.06–2.09)	0.021	1.47 (1.05–2.06)	0.025	1.82 (1.31–2.53)	< 0.001
No (ref)						
Employment status						
Not Working	0.57 (0.14–2.35)	0.437	0.33 (0.04–2.69)	0.297	0.44 (0.04–4.77)	0.503
Full/Partial work	0.78 (0.20–3.06)	0.717	0.46 (0.06–3.64)	0.461	0.59 (0.06–6.10)	0.654
Student (ref)						
Region						
North East	1.15 (0.55–2.38)	0.713	0.97 (0.50–1.91)	0.936	1.30 (0.63–2.71)	0.476
North West	0.97 (0.56–1.69)	0.916	1.36 (0.77–2.39)	0.291	1.42 (0.82–2.48)	0.213
East Midlands	1.95 (1.05–3.64)	0.035	1.15 (0.63–2.08)	0.653	1.49 (0.82–2.71)	0.190
West Midlands	1.35 (0.76–2.39)	0.311	0.97 (0.53–1.81)	0.936	1.65 (0.88–3.09)	0.115
East of England	1.69 (0.94–3.04)	0.077	1.08 (0.59–1.96)	0.806	1.37 (0.70–2.67)	0.362
London	1.28 (0.72–2.26)	0.402	1.66 (0.97–2.85)	0.067	2.06 (1.17–3.64)	0.013
South east	1.37 (0.78–2.38)	0.272	0.83 (0.45–1.52)	0.545	0.99 (0.55–1.80)	0.980
South west	1.40 (0.73–2.70)	0.316	0.76 (0.33–1.77)	0.522	1.32 (0.69–2.55)	0.401
Yorkshire & Humber (ref)						
Closest person with MI						
Self	1.52 (0.94–2.47)	0.091	2.40 (1.46–3.93)	0.001	1.57 (0.97–2.53)	0.066
Other	1.96 (1.45–2.64)	< 0.001	2.10 (1.57–2.82)	< 0.001	1.78 (1.30–2.42)	< 0.001
None (ref)	–		–		–	

*OR* Odds ratio, *CI* Confidence interval

### Effectiveness of TTC: knowledge, attitude and desire for social distance

#### Knowledge

**Fig. 1:**
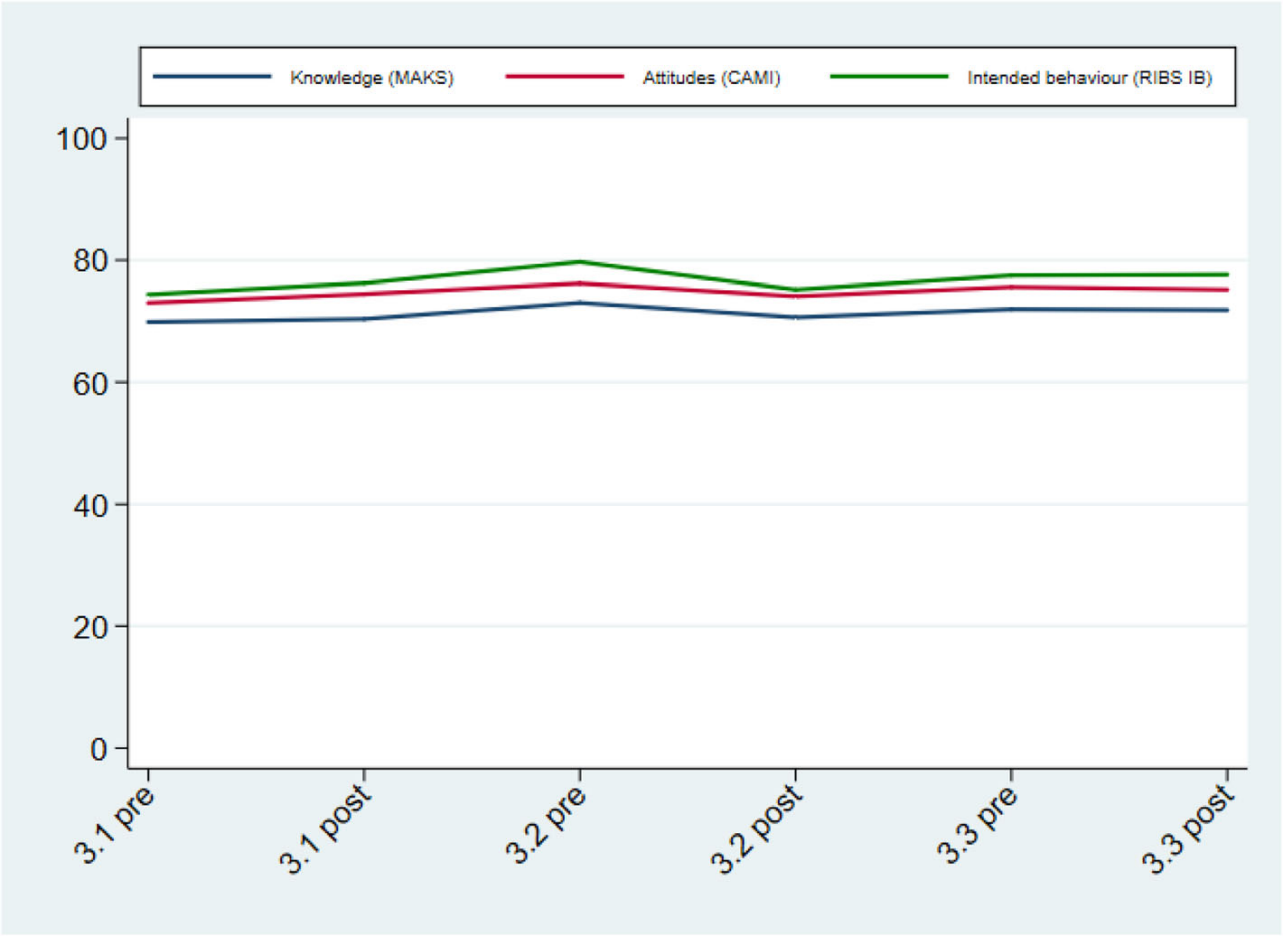
Percentage scores for the Mental Health Knowledge Schedule (MAKS) Community Attitudes toward the Mentally Ill Scale (CAMI), and Reported and Intended Behaviour Scale (RIBS) during the social marketing campaign (weighted estimates)

**Fig. 2:**
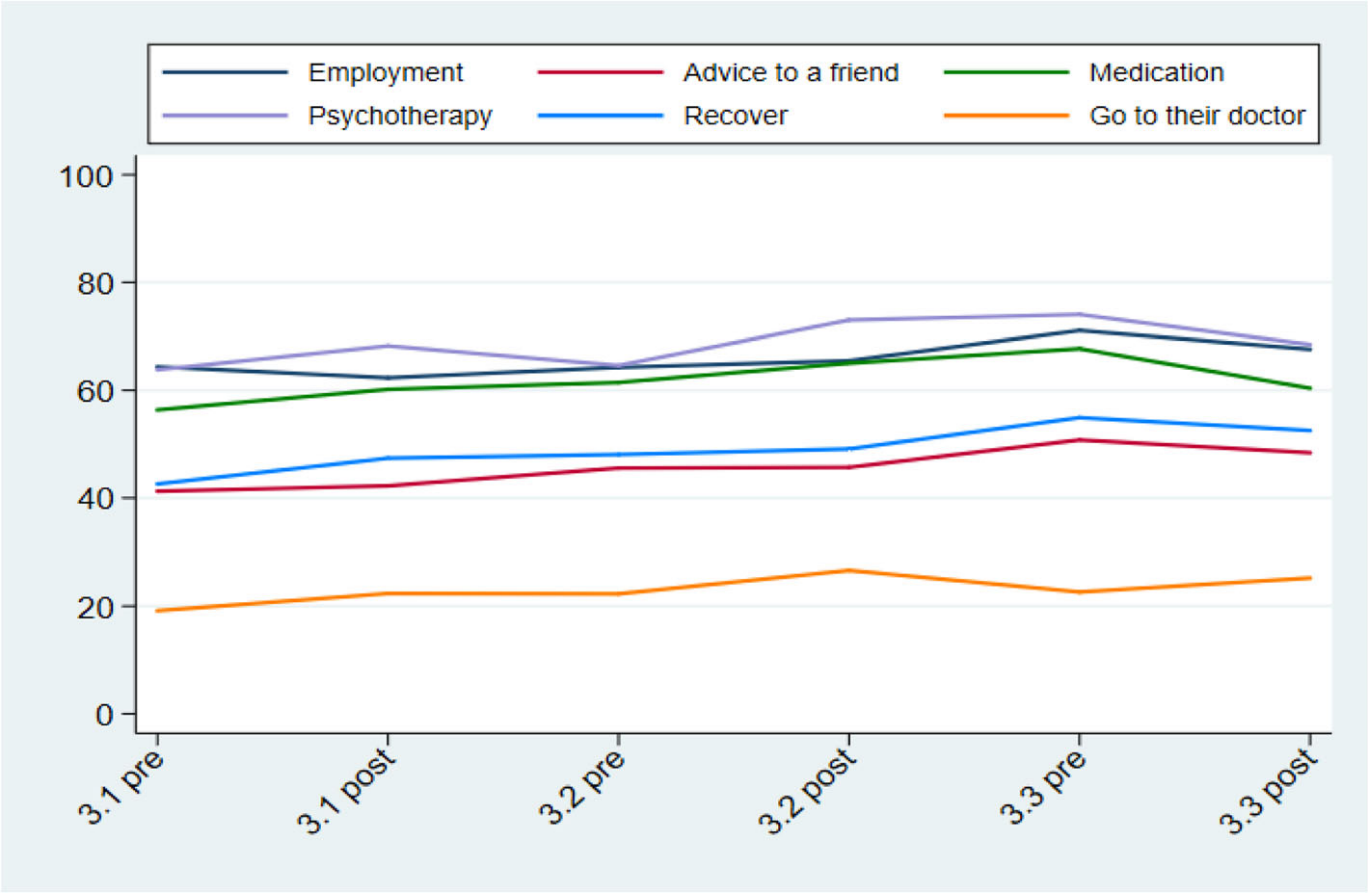
Scores of Mental Health Knowledge Schedule items during the three bursts of the social marketing campaign (weighted estimates). All items score on a 5-point Likert scale, from 5 = ‘strongly agree’ to 1 = ‘strongly disagree’. Employment: Most people with mental health problems want to have paid employment; Advice to a friend: If a friend had a mental health problem, I know what advice to give them to get professional help; Medication: Medication can be an effective treatment for people with mental health problems; Psychotherapy: Psychotherapy (e.g. counselling or talking therapy) can be an effective treatment for people with mental health problems; Recover: People with severe mental health problems can fully recover; Go to the doctor: Most people with mental health problems go to a healthcare professional to get help.

No significant pre/post differences were found in the total score of the MAKS after each of the three bursts. Over the course of all three bursts, analyses reveal a significant increase in the “Recover” item (*People with severe mental health problems can fully recover*) ((OR = 1.10, CI = 1.00 to 1.20, *p* = 0.045) and the “Advice to a friend” item (*If a friend had a mental health problem, I know what advice to give them to get professional help*) (OR = 1.10, CI = 1.01 to 1.21, *p* = 0.037), but not on any other item nor the total score. Overall percentage and item scores from the MAKS scale for each time point can be seen in Figs. 1 and 2 respectively.

Figures 1 and 2 about here.

When all three bursts were combined, campaign awareness was significantly associated with a greater MAKS score (β = 0.60, CI = 0.36 to 0.84; *p* < 0.001), Other factors associated with a greater total MAKS score were being female (β = 0.53, CI = 0.30 to 0.76; *p* < 0.001), having children (β = 0.38, CI = 0.13 to 0.63; *p* = 0.003) and having had social contact with people with mental problems (β = 1.44, CI = 1.21 to 1.67; *p* < 0.001) or experiencing them oneself (β = 2.91, CI = 2.58 to 3.33; *p* < 0.001). Asian ethnicity was associated with lower MAKS score (β = − 0.71, CI = − 1.07 to − 0.35; *p* < 0.001). Results of the linear regression model to explore factors associated with the total MAKS score, including reference categories, are presented in Table 3.

**Table 3:** Results of multivariate linear regression models to explore factors associated with MAKS, CAMI and RIBS

	MAKS (*n* = 3700)		CAMI (*n* = 3700)		RIBS (*n* = 3700)	
	β (95% CI)	*p* value	β (95% CI)	*p* value	β (95% CI)	*p* value
Burst	0.10 (−0.03 to 0.23)	0.125	0.01 (−0.30 to 0.33)	0.928	0.10 (− 0.05 to 0.25)	0.188
Awareness	0.60 (0.36 to 0.84)	< 0.001	0.30 (− 0.28 to 0.88)	0.310	0.58 (0.31 to 0.84)	< 0.001
Age						
25–29	− 0.23 (− 0.54 to 0.09)	0.160	− 0.72 (−1.48 to 0.03)	0.059	0.76 (0.41 to 1.11)	< 0.001
30–34	− 0.10 (− 0.40 to 0.19)	0.503	− 0.61 (− 1.31to 0.10)	0.093	0.45 (0.11 to 0.79)	0.010
35–39	− 0.15 (− 0.43 to 0.13)	0.290	− 0.37 (− 1.05 to 0.31)	0.281	0.38 (0.06 to 0.69)	0.019
40–45 (ref)						
Gender						
Female	0.53 (0.30 to 0.76)	- < 0.001	2.39 (1.82 to 2.96)	< 0.001	0.22 (−0.05 to 0.48)	0.111
Male (ref)-						
Ethnicity						
Black	0.11 (−0.48 to 0.70)	0.716	−0.07 (−1.46 to 1.31)	0.918	−0.90 (− 1.66 to − 0.15)	0.019
Asian	− 0.71 (− 1.07 to − 0.35)	< 0.001	−3.18 (−4.01 to −2.35)	< 0.001	− 1.15 (− 1.57 to − 0.72)	< 0.001
Mixed	0.41 (− 0.38 to 1.20)	0.309	−0.71 (− 2.60 to 1.19)	0.465	− 0.86 (− 1.75 to 0.02)	0.056
Other	− 0.80 (− 2.84 to 1.23)	0.439	−5.32 (−8.23 to − 2.41)	< 0.001	− 0.15 (− 1.66 to 1.37)	0.851
White (ref)-						
Socioeconomic status						
C2, skilled working class	− 0.23 (− 0.48 to 0.02)	0.066	− 0.87 (− 1.50 to − 0.24)	0.007	−0.02 (− 0.30 to 0.26)	0.894
D, working class	−0.20 (− 0.48 to 0.07)	0.151	− 0.90 (− 1.55 to − 0.25)	0.007	−0.15 (− 0.47 to 0.17)	0.357
C1, low-middle class (ref)-						
Married						
Yes	0.08 (−0.19 to 0.35)	0.549	−0.12 (− 0.77 to 0.53)	0.712	0.39 (0.06 to 0.71)	0.021
No (ref)-						
Children						
Yes	0.38 (0.13 to 0.63)	0.003	−0.05 (−0.65 to 0.55)	−0.872	0.16 (− 0.13 to 0.45)	0.274
No (ref)						
Employment status						
Not Working	−0.11 (− 1.45 to 1.24)	0.877	− 0.61 (−3.62 to 2.41)	0.694	− 0.87 (− 1.90 to 0.15)	0.094
Full/Partial work	− 0.36 (− 1.67 to 0.96)	0.597	− 1.63 (−4.53 to 1.27)	0.271	− 0.78 (− 1.74 to 0.18)	0.112
Student (ref)						
Region						
North East	0.17 (− 0.37 to 0.71)	0.530	1.39 (0.10 to 2.68)	0.035	0.39 (−0.19 to 0.97)	0.190
North West	0.23 (−0.20 to 0.65)	0.294	0.63 (−0.37 to 1.62)	0.217	0.11 (−0.38 to 0.61)	0.656
East Midlands	0.03 (−0.44 to 0.49)	0.915	0.47 (−0.62 to 1.56)	0.394	−0.34 (− 0.88 to 0.19)	0.210
West Midlands	0.17 (−0.29 to 0.64)	0.469	−0.18 (−1.23 to 0.86)	0.730	0.15 (−0.38 to 0.68)	0.575
East of England	0.17 (−0.30 to 0.63)	0.483	−0.77 (−1.92 to 0.38)	0.188	−0.50 (−1.05 to 0.05)	0.075
London	−0.03 (− 0.47 to 0.41)	0.887	−2.08 (−3.08 to − 1.07)	< 0.001	−0.75 (− 1.29 to − 0.22)	0.005
South east	0.08 (− 0.35 to 0.52)	0.706	− 0.30 (− 1.33 to 0.72)	0.561	−0.07 (− 0.60 to 0.46)	0.790
South west	0.23 (−0.28 to 0.75)	0.375	0.49 (−0.78 to 1.75)	0.450	−0.12 (− 0.78 to 0.54)	0.717
Yorkshire & Humber (ref)						
Closest person with MI						
Self	2.96 (2.58 to 3.34)	< 0.001	7.22 (6.40 to 8.04)	< 0.001	3.20 (2.82 to 3.57)	< 0.001
Other	1.44 (1.21 to 1.67)	< 0.001	2.92 (2.36 to 3.49)	< 0.001	2.12 (1.85 to 2.38)	< 0.001
None (ref)	–		–	–	–	

*MAKS* Mental Health Knowledge Schedule, *CAMI* Community Attitudes toward the Mentally Ill Scale, *RIBS* Reported and Social distance desire Scale, *O* Odds ratio, *CI* Confidence interval

#### Attitude

No significant pre/post differences were found in the total CAMI score after each of the bursts nor a significant improvement across all three bursts. CAMI percentage scores for each time point can be seen in Fig. 1.

When combining all three bursts, no significant association was found between campaign awareness and the CAMI total score. Factors associated with a more positive attitudes towards mental illness were being female (β = 2.39, CI = 1.82 to 2.96; *p* < 0.001), lower middle class (β = 0.90, CI = 0.25 to 1.55; *p* = 0.007), being from the North East (β = 1.39, CI = 0.10 to 2.68, *p* = 0.035) and having familiarity with people with mental problems (β = 2.92, CI = 2.35 to 3.49; *p* < 0.001) or suffering from them oneself (β = 7.22, CI = 6.40 to 8.04; *p* < 0.001). Being Asian or other ethnicity and living in London were factors associated with a lower CAMI scores (β = − 3.18, CI = − 4.01 to − 2.35, *p* < 0.001; β = − 5.32, CI = − 8.23 to − 2.41, *p* < 0.001; β = − 2.08, CI = − 3.08 to − 1.07, *p* < 0.001). Results of the linear regression model to explore factors associated with the total CAMI score, including reference categories, are presented in Table 3.

#### Desire for social distance

**Fig. 3:**
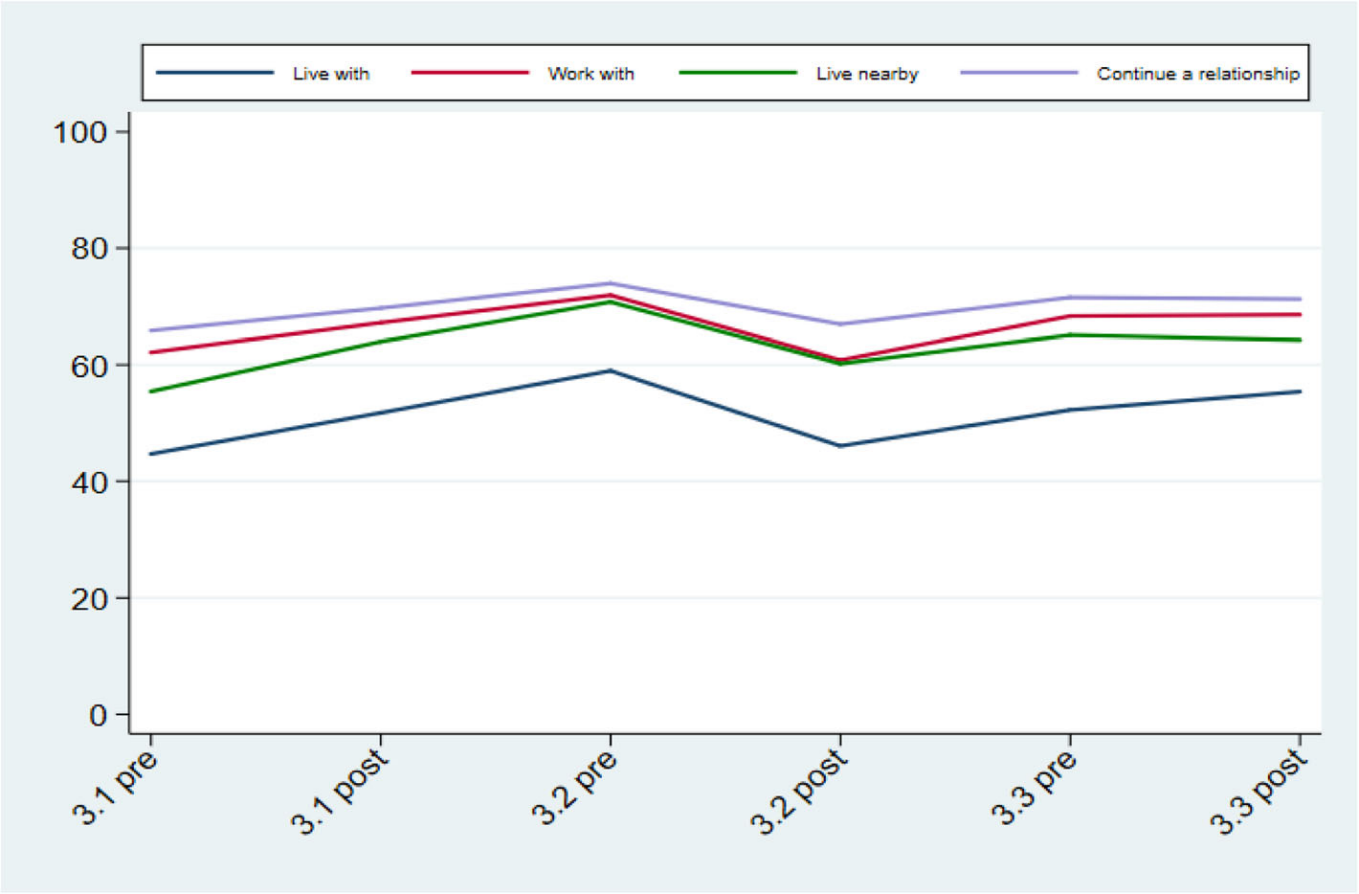
Scores of the Reported and Intended Behaviour Scale items during the three bursts of the social marketing campaign (weighted estimates). All items are score on a 5-point Likert scale, from 1 = ‘strongly disagree to engage in the stated behaviour’ to 5 = ‘strongly agree with engaging in the stated behaviour’. Live with**:** Are you currently living with, or have you ever lived with, someone with a mental health problem?; In the future, I would be willing to live with someone with a mental health problem; Work with: Are you currently working with, or have you ever worked with, someone with a mental health problem?; In the future, I would be willing to work with someone with a mental health problem; Live nearby**:** Do you currently have, or have you ever had, a neighbour with a mental health problem?; In the future, I would be willing to live nearby to someone with a mental health problem; Continue a relationship**:** Do you currently have, or have you ever had, a close friend with a mental health problem?; In the future, I would be willing to continue a relationship with a friend who developed a mental health problem.

No significant pre/post differences were found in the total RIBS intended behaviour score after each of the bursts. However across all three bursts there was a significant improvement in the “living with” (*In the future, I would be willing to live with someone with a mental health problem*) item (OR = 1.13, CI = 1.03 to 1.25; *p* = 0.008). Overall percentage and item scores from the RIBS scale for each time point can be seen in Figs. 1 and 3 respectively.

Figure 3 about here.

For all three bursts combined, there was a statistically significant positive association between campaign awareness and the total RIBS score (β = 0.58, CI = 0.31 to 0.84; *p* < 0.001). Other factors associated with the level of intended future contact with people with mental health problems are being under 40 years of age (β = 0.38, CI = 0.06 to 0.69, *p* = 0.019; β = 0.45, CI = 0.11 to 0.79, *p* = 0.010; β = 0.76, CI = 0.41 to 1.11, *p* < 0.001), being married (β = 0.39, CI = − 0.06 to 0.71; *p* = 0.021) and having had social contact with people with mental problems (β = 2.12, CI = 1.85 to 2.38; *p* < 0.001) or experiencing them oneself (β = 3.20, CI = 2.82 to 3.57; *p* < 0.001). Being Black or of Asian ethnicity and living in London were associated with lower scores in the RIBS (β = − 0.90, CI = − 1.66 to − 0.15, *p* = 0.019; β = − 1.15, CI = − 1.57 to − 0.72, *p* < 0.001; β = − 0.75, CI = − 1.29 to − 0.22, *p* = 0.005). Results of the linear regression model to investigate factors associated with the total RIBS score, including reference categories, are presented in Table 3.

## Discussion

These interim results suggest that the campaign is reaching and having some impact on its new target audience (people aged between mid-twenties and mid-forties of middle-low income groups and more focused on men), at least in terms of some domains of stigma related knowledge and desire for social distance. The results are similar to those obtained in the first phase of the campaign, with improvements only in RIBS item “living with” [13]; followed in the second phase by improvements in the “work with” and “live nearby” items of RIBS, and “paid employment”, “advice”, and “recover” items of MAKS [12].

The strongest predictive variable of knowledge, attitude and social distance desire throughout the three bursts, was having or having had contact with a person with mental health problems or suffering from them oneself. The campaign aims to promote this effect by increasing people’s confidence that they can provide supportive contact, and their desire to do so, as opposed to responding by increasing their social distance.

For each burst, moderate levels of awareness were reached, always being significantly higher in the post-measures, which indicates the transmission efficiency of the campaign. Compared to levels reached in previous campaigns (of up to 59% in 2012) [12, 13], the levels reached in this phase of the campaign are somewhat lower however, suggesting more work may be needed to identify the best methods to reach the new target group.

In our results, awareness is associated with better scores on MAKS and RIBS, but not with CAMI [12]. This may be because attitudes are a more complex construct to change, as they are strongly related to the etiological belief of mental disorder in interaction with the culture [25, 26]. Since the causality of the disease was not among the main objectives of the campaign it is possible that changes in attitudes occur more slowly and in the long term.

We found that the main factors associated with awareness are having or having had social contact with a person with mental illness, or having or having had a mental health problem oneself, and having children. Other relevant factors are being male (2nd and ^3rd^ burst), being Asian (1st and 2nd burst), and being Black or other ethnicity (3rd burst). While the results were not consistent across bursts in terms of the relationship between ethnicity and awareness, it was associated with either being Black or Asian for all three bursts. These results also seem to support the efficacy of the campaign in having focussed its content on men and adding the activities targeting parents.

While over the course of Time to Change we have found no evidence that demographic differences in stigma have widened, and indeed those by age group and region of England have narrowed, those for socioeconomic status, ethnicity and sex have so far remained unchanged [14]. By targeting a lower socioeconomic group and creating relatively greater awareness among men and in Black and ethnic minority groups, the campaign is showing the potential to address these persistent differences in stigma.

Certain limitations in the study should also be mentioned. Firstly, it is important to point out that, as self-reporting measures, evaluation can always be affected by response trends or phenomena such as social desirability, which can be accentuated by measuring a sensitive and controversial construct such as stigma. Moreover, it was not possible to randomize participants or to manipulate the intervention since this is a real-world study, and there may be variables associated with campaign awareness which are also associated with more positive attitudes. In the same way it is possible that indirect effects of the campaign will affect the results of the campaign, since there may be individuals who do not recognize the campaign but have discussed it with others. Also, a previous campaign called Heads Together could have affected some of the awareness measured, especially at the first pre point. However, the market research agency used TTC campaign materials to ascertain awareness of TTC specifically.

Finally, it is necessary to keep in mind that changes in attitudes and behaviours can occur in the longer term both positively and negatively, being phenomena in constant interaction with other influences. For instance, a participant might not have scored highly on the scales at the time of the post-burst but if a close relative suffers from a mental illness at a later stage, that same participant might act differently because of their previous experience with the campaign. However, despite the importance of long-term measures on the effects of anti-stigma programmes, few studies provide them [27].

## Conclusions

The results of the present study reveal early evidence of the effectiveness of the third phase of Time to Change anti-stigma campaign targeting a lower income group than in the previous phases and more focused on men, based on the similarity of the results to those of phase 1. The shift in content focus to men and the activities aimed at parents were effective in raising awareness of the campaign in these groups. However, it remains to be seen whether the campaign can lead to a narrowing of the pre-existing differences in stigma by socioeconomic status, ethnicity and sex. In order to address these inequalities most effectively, a better evidence base is needed regarding the reasons for these demographic differences in stigma.

## Data Availability

Data are not yet available as this is part of an ongoing study. At the end of the study they may be available on reasonable request to the senior author (Claire Henderson), subject to approval from the funders.

## Abbreviations

C1: Lower middle class
C2: Skilled working class
CAMI: Community Attitudes toward Mental Illness,
CI: Confidence interval.
D: Semi-skilled and unskilled manual workers
MAKS: Mental Health Knowledge Schedule;
OR: Odds ratio
RIBS: Reported and Intended Behaviour Scale,
UK: United Kingdom
